# Interaction of Tau with Kinesin-1: Effect of Kinesin-1 Heavy Chain Elimination on Autophagy-Mediated Mutant Tau Degradation

**DOI:** 10.3390/biomedicines12010005

**Published:** 2023-12-19

**Authors:** Karthikeyan Selvarasu, Abhay Kumar Singh, Avinash Dakshinamoorthy, Sravan Gopalkrishnashetty Sreenivasmurthy, Ashok Iyaswamy, Moorthi Radhakrishnan, Supriti Patnaik, Jian-Dong Huang, Leonard L. Williams, Sanjib Senapati, Siva Sundara Kumar Durairajan

**Affiliations:** 1 Molecular Mycology and Neurodegenerative Disease Research Laboratory, Department of Microbiology, School of Life Sciences, Central University of Tamil Nadu, Neelakudi, Thiruvarur 610 005, India; karthibiotech426@gmail.com (K.S.); rajputabhay58@gmail.com (A.K.S.); supritipatnaik3@gmail.com (S.P.); 2 Department of Biotechnology, Bhupat and Jyoti Mehta School of Biosciences, Indian Institute of Technology Madras, Chennai 600036, India; avimoorthy.1996@gmail.com (A.D.); sanjibs@iitm.ac.in (S.S.); 3 Department of Neurology, The University of Texas Medical Branch, Galveston, TX 77555, USA; sravangs@gmail.com; 4 Mr. & Mrs. Ko Chi-Ming Centre for Parkinson’s Disease Research, School of Chinese Medicine, Hong Kong Baptist University, Hong Kong SAR, China; ashokenviro@gmail.com; 5 Department of Biochemistry, Karpagam Academy of Higher Education, Coimbatore 641021, India; 6 School of Biomedical Sciences, Li Ka Shing Faculty of Medicine, The University of Hong Kong, Pokfulam, Hong Kong SAR, China; 7 Center for Excellence in Post Harvest Technologies, North Carolina Agricultural and Technical State University, The North Carolina Research Campus, 500 Laureate Way, Kannapolis, NC 28081, USA

**Keywords:** Alzheimer’s disease, tau, kinesin 1 heavy chain, KIF5B, ATPase, autophagy

## Abstract

Natively unfolded tau has a low propensity to form aggregates, but in tauopathies, such as Alzheimer’s disease (AD), tau aggregates into paired helical filaments (PHFs) and neurofibrillary tangles (NFTs). Multiple intracellular transport pathways utilize kinesin-1, a plus-end-directed microtubule-based motor. Kinesin-1 is crucial in various neurodegenerative diseases as it transports multiple cargoes along the microtubules (MT). Kinesin-1 proteins cannot progress along MTs due to an accumulation of tau on their surfaces. Although kinesin-1-mediated neuronal transport dysfunction is well-documented in other neurodegenerative diseases, its role in AD has received less attention. Very recently, we have shown that knocking down and knocking out of kinesin-1 heavy chain (KIF5B KO) expression significantly reduced the level and stability of tau in cells and tau transgenic mice, respectively. Here, we report that tau interacts with the motor domain of KIF5B in vivo and in vitro, possibly through its microtubule-binding repeat domain. This interaction leads to the inhibition of the ATPase activity of the motor domain. In addition, the KIF5B KO results in autophagy initiation, which subsequently assists in tau degradation. The mechanisms behind KIF5B KO-mediated tau degradation seem to involve its interaction with tau, promoting the trafficking of tau through retrograde transport into autophagosomes for subsequent lysosomal degradation of tau. Our results suggest how KIF5B removal facilitates the movement of autophagosomes toward lysosomes for efficient tau degradation. This mechanism can be enabled through the downregulation of kinesin-1 or the disruption of the association between kinesin-1 and tau, particularly in cases when neurons perceive disturbances in intercellular axonal transport.

## 1. Introduction

Intracellular transport, cell shape, and polarity regulation rely heavily on motor proteins, microtubules (MTs), and microtubule-associated proteins (MAPs). MAPs are responsible for stabilizing microtubule tracks, whereas motor proteins are involved in transporting cargo along the MTs. However, there is also a degree of interference between the two proteins, resulting in the MAP-mediated inhibition of the cargo movement. Kinesin motors travel toward the cell periphery, while dynein motors move towards the cell body [1]. Moreover, the activity of kinesin and dynein can be directed to specific regions in the cell by regulatory proteins. MAPs such as tau, MAP2, or MAP4 decorate MTs and facilitate the assembly of MTs [2]. Among MAPs, tau, the most abundant in neurons, plays a crucial role in preserving the integrity of axonal microtubules. Furthermore, tau can influence the movement of vesicles inside cells. An illustration of this is the study conducted by Ebneth et al. [3], which revealed that an excessive amount of tau hinders the transportation of vesicles in the direction of the plus end of MTs, thereby increasing the dominance of minus-end-directed transport. As tau inhibits kinesin’s attachment to MTs, a bias in intracellular trafficking occurs even though kinesin’s velocity is unaffected [4]. This leaves neurons susceptible to oxidative stress due to a lack of vesicles and organelles in intracellular trafficking [5].

Tau undergoes approximately three times greater phosphorylation in Alzheimer’s disease (AD) than in a normal adult brain. This hyperphosphorylation causes tau to polymerize into paired helical filaments (PHF), leading to the formation of neurofibrillary tangles (NFT) [6]. Multiple intracellular transport pathways utilize kinesin-1, a plus-end-directed microtubule-based motor [7,8]. Kinesin-1 consists of two kinesin heavy chains (KHC), forming a dimer that binds two kinesin light chains (KLCs). Cargo-binding KHCs are typically but not always associated with KLC [9]. Eukaryotic cells possess three KHCs, with KIF5A and KIF5C specific to neurons, whereas KIF5B is expressed throughout the body [2]. KHC is composed of three domains: the “head,” “stalk,” and “tail”. The “head” domain is responsible for ATP-powered anterograde transport along microtubules, the “stalk” domain assists in the dimerization of the heavy chains, and the “tail” domain governs the binding of cargo. Kinesin-1 plays a crucial role in various neurodegenerative diseases. Notably, patients with AD demonstrated elevated expression levels of genes related to the kinesin-1 family, prompting researchers to hypothesize that high expression of kinesin-1 isoforms could accelerate neuronal dysregulation in AD patients [10,11]. Recently, we have shown that the protein level of KIF5B was significantly increased in a transgenic mouse model of tau [12]. 

Accumulation of tau on microtubule surfaces prevents motor proteins from moving along them when tau is overexpressed in vivo or introduced to microtubules in vitro in excess amounts [5,13,14]. At normal concentrations, however, tau and related MAPs are helpful for motor transport by making room for microtubules surrounding them [15]. Ebneth et al. [3] showed that at relatively low levels of tau overexpression, tau retards intracellular transport of organelles and vesicles along microtubules in different cell lines. In vitro tau-decorated MT studies have demonstrated that tau acts as an impediment on the surface of microtubules, inhibiting the motility of kinesin-1 [16,17,18]. However, dynein has greater obstacle-navigation abilities and can only be blocked by large tau levels [3,16,19]. The microtubule-binding domain of tau was sufficient to reduce the motor activity of kinesin in tau-decorated microtubules [16], and this inhibition occurred at approximately one-tenth of the concentration of tau required to hinder dynein. 

Neurons rely on the lysosomal degradation system known as autophagy, which is crucial for neuronal homeostasis. The distal axon is the primary site of autophagosome formation [20]. Microtubules are involved in the association and movement of autophagosomes. They also play a role in directing mature autophagosomes toward degradation and are essential for autophagosome flux. Autophagosomes show bidirectional mobility, possibly powered by both kinesin-1 and dynein motors, for a brief period immediately following the formation of the compartment. Still, they quickly convert to robust retrograde transport throughout the length of the axon [8,21]. Microtubules and the dynein-dynactin motor complex play a vital role in the fast transport of newly formed autophagosomes toward the cell body, where lysosomes are often concentrated [22]. Mounting evidence suggests that the motor protein dynein plays a crucial role in governing the retrograde transport of autophagosomes. Since dynein is required for the retrograde transport of autophagosomes toward the cell body, downregulating kinesin may make this process easier. This could mediate autophagic flow and contribute to the breakdown of insoluble tau species by promoting the fusion of autophagosomes with lysosomes. In our recent study, we provided evidence of increased levels of KIF5B in the brains of tau transgenic mice (P301S tau mice), and we discovered that knocking down or knocking out (KO) KIF5B expression significantly reduced the level of tau in cells [12]. In addition, KIF5B restoration significantly increased both phospho and total tau in KIF5B-KO cells. We also revealed that lowering the KIF5B levels in P301S tau mice improved memory by decreasing the levels of soluble and insoluble phosphorylated tau proteins. While these findings suggest that KIF5B might be involved in tau regulation and Alzheimer’s disease (AD), the precise molecular mechanisms through which KIF5B regulates tau degradation remain unclear.

In this study, we report that tau interacts with the motor domain of KHC, possibly through its N-terminal projection domain. This interaction leads to the inhibition of the ATPase activity of the motor domain. Consequently, when KIF5B is removed, autophagy is activated, which, in turn, facilitates tau degradation. The mechanisms behind KIF5B-mediated tau degradation seem to involve its interaction with tau, promoting the trafficking of autophagosomes through dynein-dependent transport into autophagosomes for subsequent lysosomal degradation of tau.

## 2. Materials and Methods

### 2.1. Cell Lines Reagents, Recombinant Proteins, Plasmids, and Antibodies 

E. coli BL21 was purchased from Thermo Scientific (Waltham, MA, USA). Luria agar and broth B media were purchased from Difco (Franklin, NJ, USA). The glutathione sepharose 4B was purchased from GE Healthcare (Chicago, IL, USA). The Hap1-wildtype (HAP1-WT) and HAP1-KIF5B knockout (HAP1-KO) cells were purchased from Horizon (Perkin Elmer Company, Waltham, MA, USA). All the cell culture materials such as DMEM, Dulbecco’s phosphate-buffered saline, trypsin-EDTA, glutamine, fetal bovine serum (FBS), penicillin-streptomycin, and Earle’s Balanced Salt Solution (EBSS) were procured from Thermo Scientific. Chloroquine, isopropyl-1-thio-d-galactopyranoside (IPTG), paraformaldehyde (PFA), and all other chemicals, unless otherwise stated, were procured from Sigma. The mounting medium FluorSave reagent was purchased from Merck. ECL pico chemiluminescence detection kits and Roche’s protease and phosphatase inhibitor cocktail tablets were purchased from Thermo Scientific. The recombinant GST-tagged human kinesin heavy chain motor domain (GST-KHC-Motor) and recombinant human 2N4R tau protein were purchased from Cytoskeleton, Inc. (Denver, CO, USA) and Enzo Life Sciences (Farmingdale, NY, USA), respectively. The plasmid pRK5-EGFP-tau P301L (tau-P301L) was obtained from Addgene (Watertown, MA, USA). The TfLC3 plasmid (GFP-RFP-Lc3 construct) was generously gifted by Dr. T. Yoshimori at Osaka University, Japan. Antibodies: KIF5B polyclonal rabbit antibody, tau-5 mouse monoclonal antibody, LC3B rabbit polyclonal antibody, and β-actin (ACTB) mouse monoclonal were purchased from Abcam (Waltham, MA, USA), ThermoFisher Scientific, Novus Biologicals (Centennial, CO, USA), and Santa Cruz (Dallas, TX, USA), respectively. The secondary antibodies, such as AffiniPure goat anti-mouse IgG-HRP and goat anti-rabbit IgG-HRP, were procured from Jackson ImmunoResearch (West Grove, PA, USA). 

### 2.2. Affinity Isolation and Immobilization of GST-KIF5B Protein Fragments 

Following our earlier protocol [23], GST-KIF5B 1–413, GST-KIF5B 679–849, and GST-KIF5B 850–849 were purified from E. coli overexpression. At first, three different KIF5B fragments were transformed into E. coli BL21 (Waltham, MA, Thermo Scientific). Cultures of E. coli BL21 harboring the target expression vectors were cultured overnight and then diluted 1:50 in LB medium supplemented with 100 g/mL of ampicillin. Cells were grown to an optical density of 600 nm (OD_600_) of 0.7–0.8, and then induced with 0.5 mM isopropyl-1-thio-d-galactopyranoside (IPTG) to initiate the production of recombinant protein, and cultures were further incubated at 16 °C to induce the production of the soluble protein. Following overnight cultivation, the BL21 cells expressing recombinant proteins were harvested through centrifugation and stored at −80 °C. Bacterial lysis was initiated by sonicating BL21 cells harboring the recombinant protein with five cycles of 5 s on and 2 s off in lysis buffer (1 × TBS pH 7.4 with 1 mM EDTA, 1 mM DTT and supplemented with protease inhibitors such as 5 µg/mL leupeptin, 10 µg/mL aprotinin, and 10 µg/mL PMSF). The bacterial lysates were centrifugated after sonication, and the resulting supernatant was collected. Then, the bacterial lysate was incubated with 100 µL of glutathione sepharose 4B (GE Healthcare, Chicago, IL, USA) at 4 °C for 1 h with rotation at 40 rpm, after which the beads were washed in a wash buffer. To prepare GST-KHC-Motor bound sepharose beads, 100 µL of glutathione-S-sepharose beads was incubated with GST-KHC-Motor for 1 h at 4 °C, and then the beads were washed in a wash buffer. The beads were then centrifuged at 1000× *g* for 1 min three times to remove unbound proteins and blocked with the Blockace (Biorad, Hercules, CA, USA) in TBS buffer for another 30 min at room temperature, rendering them suitable for the pull-down assay. 

### 2.3. Pull-Down Assay Using Tau Mouse Brain Lysates 

The homozygous P301S mice [24] were acquired from Professor Michael Goedert (MRC Laboratory of Molecular Biology, University of Cambridge, United Kingdom). The P301S tau and wild type (C57B6) mice were maintained in the University of Hong Kong’s AAALC-accredited laboratory animal facility. All animal handling experiments, including breeding, colony care, and sacrificial procedures, were approved by the University of Hong Kong’s Committee on the Use of Live Animals in Teaching and Research (Ref #: 890-04, 1663-08, and #3399-14). Corticohippocampal tissue from 4-month-old P301S human tau or wild type mice was dissected and homogenized in 2 mL of immunoprecipitation (IP) lysis buffer (50 mM Tris 7.4, 150 mM NaCl, 1 mM EDTA, 0.5% NP-40). After homogenization, the lysates were centrifuged at 16,000× *g* for 15 min, and the supernatant was collected for further study. The lysate in the IP buffer was stirred for four hours with recombinant KIF5B fragments or GST-KHC-Motor bound to glutathione sepharose beads. After incubation, the bound beads were collected by centrifuging them at 800× *g* for 1 min. These beads were washed three times in IP buffer and once in IP washing buffer (20 mM HEPES, pH 7.4, 250 mM NaCl, 2 mM EDTA, 2 mM EGTA). Following the last wash, the proteins were eluted from the beads using 1XSDS loading buffer. The eluted proteins were then separated on SDS-PAGE, and Western blotting was performed to examine the levels of tau proteins in both the supernatants and the bound beads.

### 2.4. Pull-Down of Recombinant Tau 2N-4R by Kinesin Motor Domain

The kinesin motor protein or glutathione sepharose beads were incubated with recombinant human 2N4R tau protein (Enzo Life Sciences, Farmingdale, NY, USA) in IP buffer for 2 h at 4C with end-over-end mixing. The bound beads and supernatants were collected by centrifugation (1000× *g* for 1 min), and the beads were washed with IP washing buffer (20 mM HEPES, pH 7.4, 250 mM NaCl, 2 mM EDTA, 2 mM EGTA). Then, eluted proteins were analyzed for the presence of tau by Western blot analysis.

### 2.5. ATPase Assay

Kinetic measurements of microtubule (MT)-activated kinesin ATPase activity, with or without tau, were performed using a Kinesin Enzyme Linked Inorganic Phosphate Assay (ELIPA) kit (Cytoskeleton, Inc., Denver) as per the manufacturer’s instructions and as described elsewhere [25,26]. The assay was based on detecting a shift in absorbance, specifically from 330 nm to 360 nm, resulting from the purine nucleoside phosphorylase-mediated catalytic removal of ribonucleoside from 2-amino-6-mercapto-7-methylpurine ribonucleoside (MESG) in the presence of inorganic phosphate (Pi). Absorbance at 360 nm was directly proportional to the amount of Pi formed in the kinesin ATPase process. For kinetic measurement of the ATPase activities, the taxol-stabilized MT was mixed with GST-KHC-Motor with or without different concentrations of 2N4R tau recombinant protein (1.25 to 10 µM) and incubated for 30 min at room temperature. Each reaction contained 94 nM KIF5B motor domain protein, 0.66 μM taxol stabilized microtubules, 0.2 mM MESG, 0.3 U PNP, 15 μM taxol, 15 mM PIPES pH 7, 5 mM MgCl2, 0.6 mM ATP, and 2N4R recombinant tau protein (2.5 to 10 µM). Reactions without motor protein, MTs, or tau served as controls. After adding 60 µM of ATP, the kinetic measurement of ATPase activity was monitored by quantifying the amount of released inorganic phosphate (Pi), which was calculated using a standard curve of inorganic phosphate. A Spark^®^ multimode multiwell plate reader (Tecan, Männedorf, CH-ZH, Switzerland) with kinetic mode and 360 nm absorbance wavelength was used to measure reactions. Measurements were recorded at room temperature, with readings taken every 30 s over a continuous period of 20 min. Kinesin’s linear ATP turnover rate was determined by dividing the number of phosphate molecules released (Pi) by the total number of kinesin molecules and the elapsed time.

### 2.6. Docking of KIF5B Motor Domain with Tau

The KIF5B motor domain structure was taken from the protein data bank PDB ID: 1BG2 [27]. All the available crystal structures of tau protein were only of the tau filaments and not of the full unstructured tau 441 2N4R isoform. Therefore, the tau 441 2N4R isoform was obtained from PDB-Dev ID: pdbdev_00000033, which used structural proteomics experimental data and all-atom discrete molecular dynamics (DMD) simulations to model the structure [28]. The ClusPro web server was utilized to perform protein–protein docking with balanced score coefficients [29]. The ClusPro server performs rigid body docking followed by clustering and energy minimization. The best model from the ClusPro server based on the cluster score was chosen for binding energy prediction using the Prodigy web server [30]. The interacting residues at the interface of KIF5B and tau protein were determined using the Protein–Ligand Interaction Profiler [31].

### 2.7. Cell Culture

HAP1-KO and HAP1-WT cells were cultured in DMEM with 10% FBS containing 1% PS, as described previously [12]. In starvation experiments, after two washes in PBS, cells were starved by adding EBSS and incubating them for four hours at 37 °C in a 5% CO_2_ incubator. For plasmid transfection experiments, HAP1-WT or HAP1-KO cells were transfected with the tau-P301L plasmid or tf-LC3 plasmid using Lipofectamine 2000 according to the manufacturer’s instructions. Unless otherwise stated, assessments in in vitro experiments were performed 24 h following the transfection of the cells. After the experiments, the cells were solubilized in RIPA lysis buffer supplemented with protease and phosphatase inhibitors.

### 2.8. Cell Imaging and Puncta Counting

HAP1-WT or HAP1-KO cells transfected with tf-LC3 plasmids were cultured on 24 mm coverslips within a 24-well plate. After incubating them with or without EBSS buffer for 4 h, the cells were fixed in 4% PFA for 10 min and then mounted with FluorSave reagent. The quantification of GFP or RFP puncta in cells was performed following the previously described method [32]. A minimum of 20–25 cells were randomly chosen from each group and counted manually to determine how many red, green, or yellow puncta were present. The data presented were compiled from at least three separate experiments.

### 2.9. Analysis of Protein Lysates and Pull-Down Samples by Western Blotting

Immunoblot analysis was performed following the protocol described in our previous study [12]. The cell and brain lysates and the pull-down samples were centrifuged at 10,000× *g* for 15 min, and the resulting supernatants were then separated on 10% or 15% SDS-PAGE gels. Following electrophoresis, the proteins were transferred onto PVDF membranes for blotting. For the detection of tau, KIF5B, LC3-II and β-actin proteins, the membranes were blocked with 5% skimmed milk in 0.2% Tween 20 in Tris-buffered saline (TBST) overnight at room temperature for 2 h and then probed with specific primary antibodies, including tau-5 (1:1000), KIF5B (1:1000), LC3 (1:10,000), and β-actin (1:1000), respectively. After overnight incubation, the blots were washed in TBST and incubated with the appropriate secondary antibodies at room temperature for 1 h. The desired chemiluminescent signals were captured using an X-ray film in a dark room using the ECL kit. The density of signals was semi-quantified using the Image J software (1.8.0_345).

### 2.10. Statistics

The data were expressed as mean ± standard error of the mean (SEM). One-way ANOVA was used for multiple comparisons, followed by the Newman–Keuls multiple post hoc comparisons test. The data were graphically represented and analyzed with GraphPad Prism 9 and considered statistically significant at *p* < 0.05 (*), *p* < 0.01 (**) and *p* < 0.0001 (****).

## 3. Results

### 3.1. KIF5B Motor Domain Interacts with Tau

**Figure 1:**
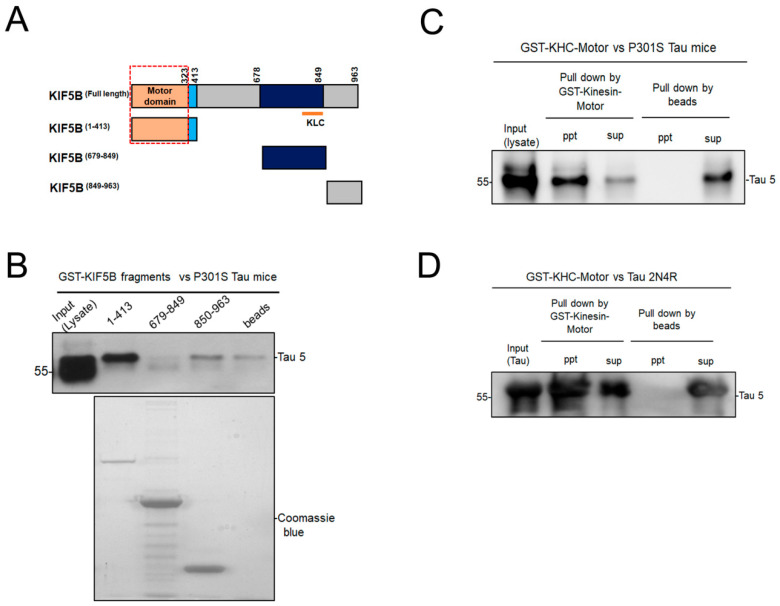
Kinesin-1 motor domain interacts with tau in vivo and in vitro. (**A**). Diagrammatic representation of KIF5B fragments and the motor domain of KIF5B is indicated within the red frame. (**B**). Western blot of GST-tagged KIF5B fragments (motor + stalk, neck and tail region) with pull down of tau from P301S tau mouse brain lysate and the brain lysate was loaded as input. The purity of KIF5B fragments was ascertained by Commassie blue staining of SDS -PAGE gel (lower panel). (**C**). Western blot of GST-KHC-Motor with pull down of tau from P301S tau mouse brain lysate. (**D**). Western blot of GST-KHC-Motor with pull down of recombinant 2N4R tau protein. The tau levels in the precipitates (ppt) and supernatants (sup) of samples loaded with GST-Kinesin motor coupled beads or beads alone were demonstrated.

KIF5B consists of three functional regions: the motor, stalk, and tail domains. The tail domain of KIF5B is responsible for its interaction with the kinesin light chain (KLC) [33]. To investigate which domain of KIF5B interacts with tau, KIF5B fragments fused with the GST tag in the N-terminal were used as described previously [23]. These fragments were overexpressed in the E. coli BL21 strain, and the recombinant proteins were immobilized on glutathione sepharose beads. Then, these agarose beads were used to pull down KIF5B-interacting proteins from the P301S tau mouse brain lysate. KIF5B motor domain with a hinge region, 1 to 413 in amino acid sequence, was able to pull down tau, while fragments containing light chain binding domain (679–849) and tail region (850–963) had no pull-down effect (Figure 1A,B). Thus, KIF5B interaction with tau depends on the KIF5B motor domain and is light chain independent. To identify whether the kinesin motor domain (1–323) binds with tau in vivo, we employed GST-KHC-Motor to pull down tau from the P301S tau mouse brain lysate. Our results showed that the kinesin motor attached to tau firmly in the precipitates of GST-KHC-Motor loaded beads. In contrast, tau could not be pulled down by beads alone (Figure 1). The identification and purity of the GST-KHC-Motor protein were ascertained through the Western blotting and SDS-PAGE techniques, respectively. The observed level of purity exceeds 85% when compared to the band profiles mentioned in the datasheet (Figure S1). Next, we attempted to pull down tau from both the WT and P301S tau mouse brain lysates using GST-KHC-Motor beads. The results indicated that tau protein was not identifiable in the brain lysates of WT mice. However, a considerable amount of tau protein was successfully pulled down from P301S tau mouse brain lysate by GST-KHC-Motor (Figure S3). To investigate the interaction of kinesin motor and tau in in vitro, we performed the GST pull-down assay again using GST-KHC-Motor and recombinant tau 441 2N4R protein. Our findings indicate that the motor domain of kinesin interacted with the tau protein. (Figure 1C). However, tau did not interact with the glutathione beads alone, demonstrating the assay’s specificity. (Figure 1C). To further confirm GST-KHC-Motor interaction and tau interaction in vivo and in vitro, we analyzed the tau level in the supernatants of GST-KHC-Motor and bead-alone samples. As expected, there were reduced tau levels in the superannuants of the GST-KHC-Motor precipitated sample compared to beads alone (Figure 1B,C). Taken together, these results imply that, unlike the other KIF5B domains, the motor domain and tau interact directly with one another.

### 3.2. Tau Inhibits the Kinesin Motor’s Intrinsic ATPase Activity

**Figure 2:**
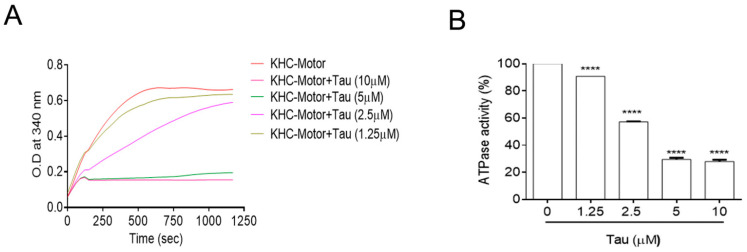
Regulation of the motor activity of KHC-Motor by recombinant tau 441 2N4R (**A**). The ATPase activity of the KHC-motor (50 nM) is stimulated by microtubules (MT; 300 nM) in ELIPA assay, which was performed with or without different concentrations of recombinant tau (1.25 to 10 μM). (**B**). The increasing concentrations of tau 2N4R inhibit the ATPase activity of the kinesin motor from 0 to 5 min. The data were presented as the mean± SEM from three separate experiments. **** *p* < 0.0001 vs. KHC-Motor alone.

Using ATPase assay for the kinesin motor domain with or without microtubules, we investigated whether tau influenced the ATPase activity of the KHC-Motor (Figure 2A). The ATPase function of the kinesin motor domain is increased in the presence of microtubules from 30 to 1200 s (Figure 2A). However, this kinetic curve flattens out with less pronounced ATPase activity after treatment with recombinant 2N4R tau at concentrations from 1.25 to 10 µM, indicating possible interaction of tau with the ATPase catalytic site of the kinesin motor domain. To calculate the ATP turnover rate of kinesin in the same end-point assays, the assay kinetics were calculated between 5 and 8 min after adding ATP with or without different concentrations of tau. (Figure 2B) The tau protein significantly inhibited the ATPase activity of kinesin in a dose-dependent manner. As the tau concentrations increased, a significant reduction was observed in the ATPase activity of kinesin.

### 3.3. Docking

**Figure 3:**
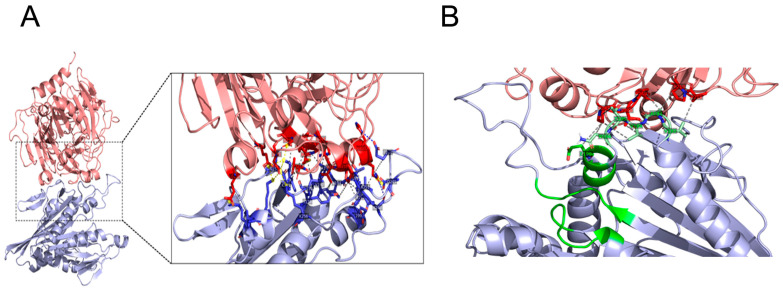
(**A**). Model of tau 2N4R-KIF5B complex from protein–protein docking. Tau protein is depicted in light red, and KIF5B in light blue. The interacting residues of tau and KIF5B are shown in the inset as licorice in red and blue, respectively. Hydrophobic, H-bond, and salt-bridge interactions are depicted as grey, blue, and yellow dashed lines, respectively. (**B**). Tau protein interacting with residues around Switch-1 motif of the ATP binding domain of KIF5B. Tau protein is depicted in light red and KIF5B in light blue. Tau protein residues interacting around the ATP binding region of KIF5B are depicted in dark red. The ATP binding domain of KIF5B is depicted in green, and its adjoining residues that interact with tau protein are shown in licorice-light green. Hydrophobic interactions are depicted as grey dashed lines.

The full-length KIF5B protein is 963 amino acids long, and it has several domains, which include the motor domain (1–324), the coiled-coil domain (325–914), and the globular domain (914–963) (Figure 3A). The coiled-coil domain can also be further classified into the hinge region (325–413), the stalk region (414–679), and the tail region (680–913), all of which are made of alpha helices arranged in a coiled-coil fashion (Figure 3A). However, the crystal structure is available only for the motor domain of the KIF5B protein (PDB ID- 1BG2) [27]. The structure of the unstructured full-length tau 2N4R was obtained from PDB-Dev (pdbdev_000033). This structure was developed using a de novo protein structure determination method using experimental cross-linking data and discrete molecular dynamics [28].

The tau-KIF5B complex was obtained by docking them together using the ClusPro web server [29]. The protein–protein docking was performed using a balanced scoring coefficient in the ClusPro server, and the model with the best cluster score was chosen as the tau-KIF5B complex structure. The binding affinity of the complex was then obtained using the Prodigy web server [30], and the interacting residues at the tau-KIF5B complex interface were obtained from the Protein–Ligand Interaction Profiler server [31]. The docking results show that the KIF5B motor region binds to tau protein with a binding affinity of ΔG = −13.00 kcal/mol. Closer inspection of the interface residue between KIF5B and tau reveals that the binding is driven mainly by certain charged and hydrophobic residues on the surface of both proteins. The tau protein interacts mainly with the KIF5B residues exposed to solvent (Figure 3B). Some tau protein residues also interacted with regions close to the ATP binding domain of KIF5B (Table S1). The ATP binding domain of the kinesin motor protein consists of several motifs that are common to myosin and G-protein, namely the Walker A motif (GXXXXGKT) (residues 85–92), switch-1 (NxxSSR) (residues 198–203) and the Switch-2 motif (DxxGxE) (residues 231–236). The switch-1 and switch-2 motifs change their conformation and interaction pattern with adjacent structural elements upon sensing the gamma-phosphate of the ATP molecule [34]. Residues E199, S202, R203, E234, and E236 of KIF5B are reported to be involved in the sensing of gamma-phosphate of ATP [32,35]. From our protein–protein complex structure, we observed that the tau protein interacts with KIF5B residues that are in switch-1 (E199) and surrounding residues 192–196 (Figure 3D). This interaction of tau protein at the ATP binding site of KIF5B can explain the dose-dependent ATPase activity inhibition of KIF5B by tau protein.

### 3.4. KIF5B KO Facilitates Autophagosome Maturation

**Figure 4:**
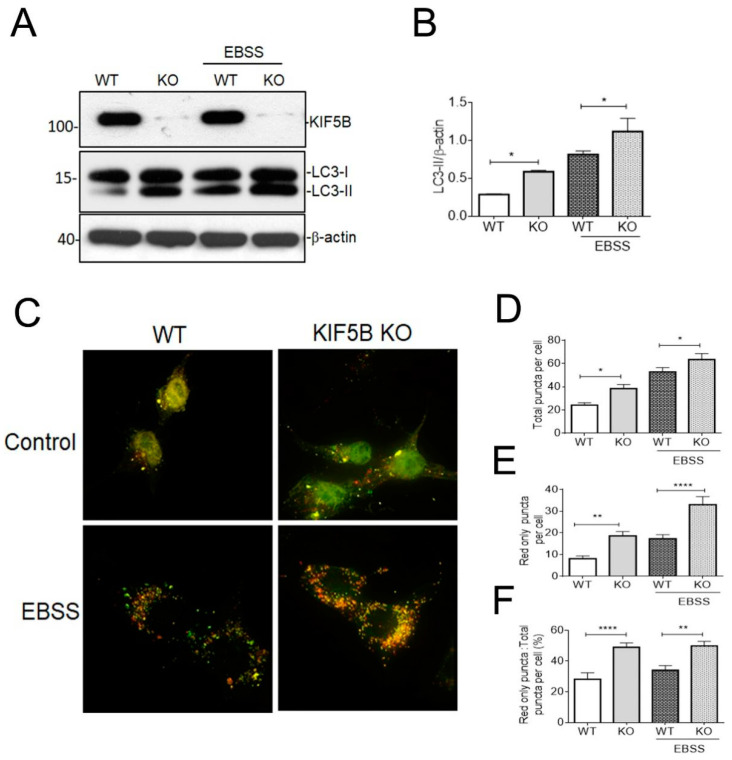
Removal of KIF5B facilitates autophagosome maturation. (**A**). Western blotting validation of knockout of KIF5B (KIF5B KO) and its effects on the autophagic marker in HAP1 cells by analyzing the level of KIF5B and LC3-II levels in HAP1-WT and HAP1-KIF5B-KO cells. KIF5B and LC3-II levels were also determined by Western blotting under starvation (EBSS) conditions both in HAP1-WT and HAP1-KIF5B-KO. (**B**). The results are represented as mean ± SEM of three independent experiments: * *p* < 0.05, vs. HAP1-WT cells (control). (**C**). Autophagosome maturation in HAP1-WT and HAP1-KIF5B-KO was determined by the tf-LC3 plasmid in normal and starvation conditions using a Deltavision fluorescent deconvolution microscope. (**D–F**). The counting data were presented as the mean ± SEM, *n* = 20–25 cells from three separate experiments ** *p* < 0.01, **** *p* < 0.0001 vs. HAP1-WT cells.

In our recent study, we have revealed a connection between KIF5B and its involvement in the regulation of tau degradation [12]. This association became evident due to the substantial reduction in the half-lives of tau observed in KIF5B KO cells compared to their wild type counterparts. The tau level was probed by the tau 5, a pan tau antibody that can detect both the phosphorylated and non-phosphorylated isoforms [36]. We also demonstrated that the level of LC3-II, which correlates with autophagosome number, was increased in the heterozygous KIF5B knockout mice [12]. These outcomes suggest a crucial function for KIF5B in modulating autophagosome trafficking and maturation in order to suppress tau degradation. We therefore analyzed HAP1-KIF5B-KO and HAP1-WT cells to determine the part KIF5B plays in controlling autophagosome production and maturation. The knockout of KIF5B augmented the levels of LC3-II compared to WT cells (Figure 4A,B). To increase autophagy flux, we starved the cells in EBSS, which led to a higher level of LC3-II in KIF5B KO cells but not in WT cells (Figure 4A,B).

Autophagosome maturation can be tracked using the Tf-Lc3 plasmid, which operates on the concept that GFP is more readily dulled than RFP at acidic pH levels in the lysosome [37]. We transiently transfected HAP1-KIF5B-KO and HAP1-WT cells with tf-Lc3 plasmids to establish the involvement of KIF5B in modifying autophagosome maturation and observed that KIF5B KO leads to the increased red punctate (autolysosomes) compared to the green punctate (autophagosomes) (Figure 4C). Interestingly, more red-only punctate were seen in HAP1-KIF5B-KO cells than HAP1-WT cells after autophagy was induced by EBSS-mediated starvation (Figure 4A). More autophagosomes fused with lysosomes were observed in HAP1-KIF5B-KO cells than in HAP1-WT cells, as evidenced by an increase in the total number of puncta (Figure 4D), the number of red-only puncta (Figure 4E), and the ratio of red puncta to total puncta (Figure 4D). Further, more autophagosomes fused with lysosomes in HAP1-KIF5B-KO cells than in HAP1-WT cells, as evidenced by an increase in the overall number of puncta and the number of red-only puncta (Figure 4C,D). These data together demonstrate that KIF5B KO facilitates autophagosome maturation, suggesting the promotion of autophagic flux by losing KIF5B. Collectively, these results show that the loss of KIF5B promotes autophagic flux probably by favoring the anterograde trafficking of autophagosome toward the cell center where the autophagosome fuses with autolysosome.

### 3.5. Removal of KIF5B Reduced Tau via Induction of Autophagy

**Figure 5:**
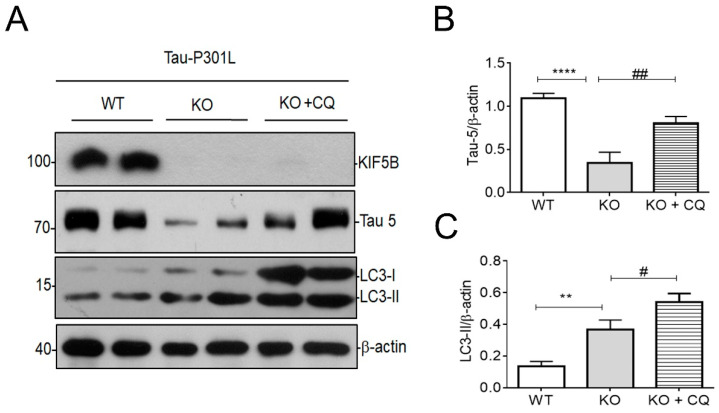
Involvement of autophagy for KIF5B KO-mediated regulation of tau levels. (**A**). The effect of CQ on the KIF5B KO-mediated degradation of tau was examined; LC3-II and β-actin were employed as markers for autophagosomes and internal control, respectively. (**B**). Semi-quantitative analysis showed that CQ blocked the KIF5B KO-mediated reduction in tau levels and autophagic flux (**C**). The data were presented as the mean± SEM from three separate experiments. ** *p* < 0.01 and **** *p* < 0.0001 vs. HAP1-WT cells and # *p* < 0.05 and ## *p* < 001 vs. HAP1-KIF5B-KO cells.

To determine whether autophagy is involved in KIFB-KO-mediated tau degradation, we monitored the level of tau and LC3-II in HAP1-KIF5B-KO and WT cells overexpressing p301L tau plasmid. As expected, we found that total tau decreased significantly in KIF5B KO compared to WT cells, even with the overexpression of P301L at 24 h (Figure 5A–C). However, we found that the KIF5B KO-mediated reduction in tau was attenuated by chloroquine (CQ), an autophagic inhibitor, treatment in HAP1-KIF5B KO cells (Figure 5A,B), indicating that the degradation of tau in KIF5B KO cell was mediated by autophagy. Additionally, we found that the endogenous LC3-II was increased significantly in KIF5B KO compared to WT cells, even with the overexpression of P301L at 24 h (Figure 5A,C). The increase in LC3-II was more significant in KIF5B KO cells with CQ to inhibit lysosomal acidification. Thus, the clearance of tau in KIF5B KO cells was found to be dependent on the autophagic flux process because the lysosome inhibitor CQ abolished the pro-clearance activity of KIF5B KO.

However, there was only a slight increase in the tau levels after HAP1-WT cells were treated with the CQ, though CQ significantly increased the LC3-II levels in HAP1-WT cells (Figure S3).

## 4. Discussion

Several neurodegenerative disorders are linked to problems associated with microtubule-based motor transport. The present study revealed an intricate relationship between KIF5B and tau protein. We found that tau interacts with KIF5B both in the brain lysates of P301S tau mice and in the in vitro assay system. KIF5B interacts with tau via its motor domain. When MTs and kinesin were coincubated, and then htau440 was added, the ATPase activity was decreased by tau in a dose-dependent manner. Finally, we showed that the knockout of KIF5B induced autophagy, resulting in the degradation of tau aggregates, which corroborates with our recent finding that the increased level of autophagy marker LC3-II increased in the P301S tau mice with heterozygous knockout of KIF5B [12]. Our results illustrate how kinesin-1 removal facilitates the movement of autophagosomes toward lysosomes. This process can be promoted by either inhibiting the expression of kinesin-1 or disrupting the interaction between kinesin-1 and tau when neurons detect a hindrance in intracellular transport. We used P301S tau mice and P301L tau plasmid for in vivo and in vitro experiments, respectively. The P301L and P301S mutations are located on exon 10 of the tau gene and can potentially induce frontotemporal dementia (FTD) [38]. Since the tau protein with P301S mutation significantly hinders its capacity to facilitate microtubule assembly and axonal transport is drastically reduced in P310S tau mice [39], we subjected P301S mice brain lysate to pull down the tau using the KHC-Motor domain. In our cell culture experiments, we used the P301L tau plasmid due to its capacity to significantly enhance the generation of paired helical filaments, which have the propensity to accumulate over time and have been observed to exhibit a degree of resistance to autophagy-mediated degradation [40].

In kinesin, post-translational alterations to the cargo-binding domain of the light and heavy chains have been shown to influence transport, but how modifications to the motor domain affect transport needs to be better understood. The precise molecular mechanism behind the interference between tau and the kinesin motor has been debated [25,41,42]. Kinesin-1 motility is inhibited in vitro and in vivo when microtubules are decorated with tau or other MAPs. These proteins have similar C-terminal microtubule-binding repeats [43,44]. Overexpression of tau in vivo or adding large amounts of tau to microtubules in vitro causes tau to accumulate on microtubule surfaces and blocks the transport of motor proteins [5,13]. Given that both motor proteins and tau interact with the microtubule surface, it is intriguing to speculate whether tau directly interacts with the motor domain of the KHC and interferes with its movement along the microtubule. In a co-decoration docking study using crystallography, Marx et al. [34] found a partial overlap of the binding sites between tau and KIF5B on microtubules; thus, tau can inhibit the movements of motor proteins in vitro. However, the exact location of the interaction between tau and the motor domain of KIF5B remains unknown. Here, we show through docking study that the KIF5B motor switch-1 region (E-199) interacted with the tau R1 repeat (P-247), which is found in the microtubule-binding region of tau. (Figure 3; Table S1). 

However, there is still a lack of information about how tau inhibited the movement of the KIF5B motor. Here, it is found that tau interacts with the kinesin-1 motor domain because the interaction site of KIF5B is narrowed down to 1 to 323 (Figure 1C), suggesting that tau may inhibit the transportation of KIF5B. This interaction is believed to inhibit kinesin-1 motor function probably by inhibiting the ATPase activity of the motor domain. Our studies are the first to show the ability of tau protein to inhibit the ATPase activity of KIF5B by directly interacting with the motor domain of KIF5B, validating the regulatory role of tau in the function of KIF5B in a biochemically defined cell-free system. Kinesins take 8 nm-sized steps as they make their way from the microtubule’s negative to the positive pole [45]. Since the mechanical and chemical (ATP hydrolysis) cycles are coupled in a one-to-one fashion [46], the rate of kinesin can be calculated as v = *kcat ATPase X* ( *kATPase*, rate of ATP hydrolysis by motor, *X* a distance moved by the kinesin per kinetic cycle) [26,46]. Based on the maximal ATPase rate that we measured in our assay ofKHC-Motor in the presence of microtubule (38 ATP/s/kinesin molecule), we estimate the speed to KHC-Motor to be around 304 nm/s. This value is still approximately one-fold higher than observed in vitro with the KIF5B full length protein [26] but threefold lower than the recombinant Drosophila kinesin-1 [47,48]. Compared to the speed of KIF5C in vivo [49] and the anterograde transport rate by kinesin 1 in squid axoplasm [50], our value is roughly 2.5 and 5-fold lower, respectively. These disparities imply that additional factors are necessary to enhance further the motor activity of KIF5B [51].

Recently, we have shown that KIF5B KO significantly decreases the half-life for tau [12]. Subsequently, we examined the connections between autophagy and the degradation of tau mediated by KIF5B. During retrograde transport down the axon, the motor proteins dynein and kinesin-1 are found to remain firmly attached to autophagosomes. Rapid retrograde movement of autophagosomes along the axon is caused by the binding of the scaffolding protein JIP1 to LC3, which prevents the activation of kinesin-1 on these organelles [52]. Given that dynein molecular motors are pivotal for both autophagosome transport and the facilitation of autophagic flux, we questioned whether KIF5B also contributes to the regulation of autophagosome maturation. We used the CRISPR-Cas9-engineered HAP1-KIF5B KO model to investigate this question. Our results showed that LC3-II levels were increased in KIF5B knockout under starvation conditions compared to wildtype. KIF5B KO stimulates autophagosome fusion with the lysosome, as evidenced by tf-LC3 labeling and autophagic flux. We found that inhibition of lysosomal activity with CQ reversed KIF5B KO-induced degradation of tau (Figure 5). In particular, KIF5B KO led to a rise in lysosomal abundance, suggesting that tau may be more efficiently delivered to the lysosome for destruction without KIF5B (Figure 4 and Figure 5). These findings provide credence to the notion that autophagy plays a role in the degradation of tau facilitated by KIF5B knockout. These findings also align with prior studies highlighting the essential role of autophagy in regulating the degradation of tau. This study’s findings provide new insight into the function of KIF5B in regulating autophagy-lysosome fusion. Maday et al. [8] discovered that both dynein and kinesin stay linked to the axonal autophagosomes, even though it is established that autophagosomes in cortical neurons undergo retrograde transportation along the axon through dynein dependency [8,53]. There are conflicting reports [54,55,56], even though we and others have found that kinesin is a competitor in autophagosome trafficking (Figure 4) [8,52]. Nevertheless, our findings have shown that KIF5B KO plays a significant role in controlling the retrograde transport of autophagosomes. Dynein-mediated autophagosome trafficking may have been accelerated in the absence of KIF5B. As shown in the diagram (Figure 5), this process may have resulted in the degradation of tau by autophagy. Lysosomes mediate the clearance of cellular substrates from the autophagic and endocytic processes. Only a small fraction of lysosomes are immobile in healthy neurites, and most of them travel in either an anterograde or retrograde direction [57]. Suppression of motor protein activity also altered the autolysosomal transport in the direction of their origin. Based on our findings, RFP-LC3-labeled autolysosomes vary in size at 4 h post-starvation, with large and small fluorescent structures often present within the HAP1-KIF5B KO cells. Therefore, stringent regulation of autophagosome or autolysosome retrograde axonal trafficking is required to maximize dynein motor activity, either by compensating for the absence of KIF5B or reducing the influence of associated kinesin activities.

## 5. Conclusions

MAPs have been shown to influence vesicle mobility in addition to their MT stabilizing activity. Tau protein binds to the inner surface of MTs after they have been polymerized with tubulins, thereby stabilizing the MTs’ structure [58]. On the other hand, if abundant tau molecules are supplied after MTs have been stabilized, they can impede kinesin movement by binding to the MTs’ outer surface [25,59,60]. The binding of tau along microtubules and the subsequent modulation of motor interaction with the microtubule surface may be a mechanism by which intracellular transport is regulated. Chaudhary et al. [61]. demonstrated that the regulation of cargoes, such as phagosomes and endosomes, which were transported by kinesin-1 and dynein, was primarily influenced by tau. This regulation was mainly mediated through kinesin-1 due to its increased sensitivity to tau. Despite tau’s ability to reduce kinesin 1 mobility, it has been shown that kinesin still displays diffusive movement on microtubule without expending energy (ATPase inhibited state) [62]. This is just one of many mechanisms by which the cell maintains its ability to transport organelles and vesicles continuously. This mechanism may facilitate the ability of kinesin to pick up cargo, and/or allow the kinesin/cargo complex to stay bound after encountering obstacles. Hence, we strongly speculate that kinesins transport the autophagosomes during the ongoing tug-of-war with dynein. Thus, eliminating this faulty KIF5B would enhance bidirectional motility toward the microtubule minus-end or the cell center, thereby promoting the effective degradation of tau aggregates.

## Data Availability

The authors of this work are willing to provide supporting data upon reasonable request.

## Supplementary Materials

The following supporting information can be downloaded at: https://www.mdpi.com/article/10.3390/biomedicines12010005/s1, Figure S1: Assessing the purity and identity of recombinant GST- Kinesin-Motor (1-323) domain by SDS-PAGE/Commassie Brilliant Blue staining (CBB) and Western blotting (WB); Figure S2: KHC-Motor protein pull downed tau from P301S Tau mouse brain lysate but not from WT mice brain lysate; Figure S3: Tau levels were determined by western blotting with or without CQ treatment in HAP1-WT cells; Table S1: All interacting residues that are involved in hydrophobic, H-bonding, and salt-bridge interactions at the Tau-KIF5B interface.
