# Supplementary material for: Interaction of Tau with Kinesin-1: Effect of Kinesin-1 Heavy Chain Elimination on Autophagy-Mediated Mutant Tau Degradation
Source: Biomedicines. 2023 Dec 19;12(1):5. doi: 10.3390/biomedicines12010005 (PMC10813313; doi:10.3390/biomedicines12010005)
Supplement: Supplementary file 1 [file biomedicines-12-00005-s001.zip › biomedicines-2605794-supplementary.pdf]

Supplementary Figures

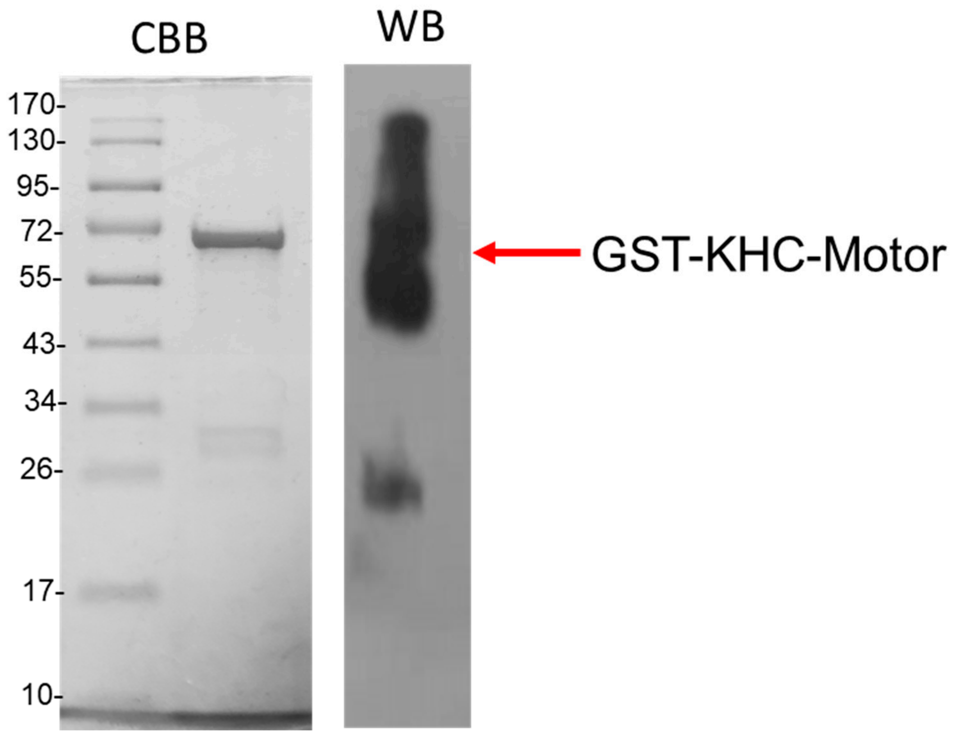

Suppl.Figure S1. Assessing the purity and identity of recombinant GST-Kinesin-Motor (1-323) domain by SDS-PAGE/Commassie Brilliant Blue staining (CBB) and western blotting (WB). The lower bands indicate the degradative product of the recombinant protein.

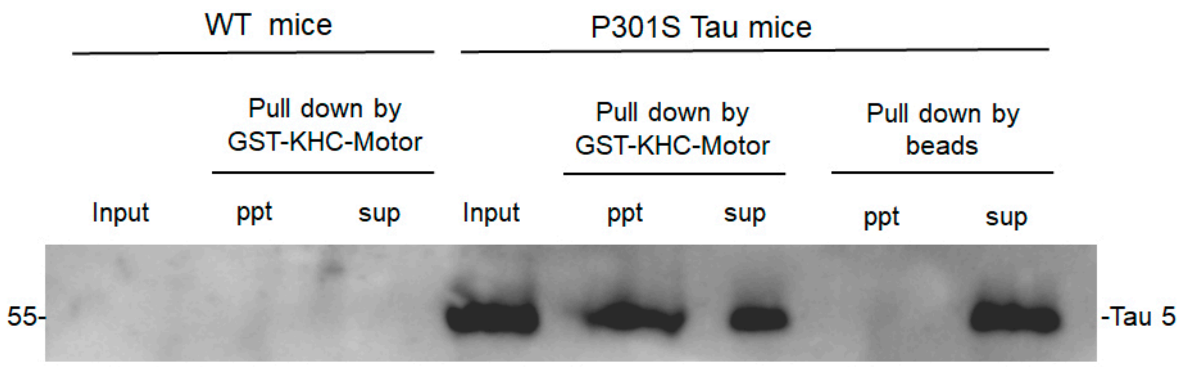

Suppl.Figure S2. KHC-Motor protein pulldowned tau from P301S Tau mouse brain lysate but not from WT mice brain lysate.

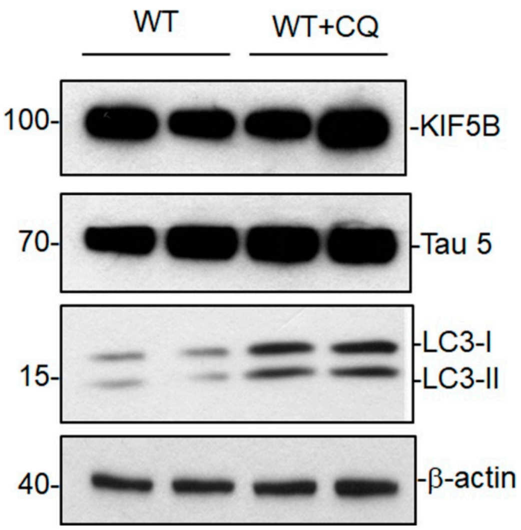

Supply.Figure S3. Tau levels were determined by western blotting with or without CQ treatment in HAP1-WT cells.

Supplementary Table S1: All interacting residues that are involved in hydrophobic, H-bonding, and salt-bridge interactions at the Tau-KIF5B interface

**Hydrophobic interactions**	
**KIFB Residue**	**Tau Residue**
TYR-138	VAL-248
TYR-138	VAL-248
LEU-139	GLN-244
LYS-141	MET-250
LYS-141	ASP-252
LYS-141	THR-414
VAL-148	PRO-218
VAL-192	PRO-218
ALA-193	PRO-249
ALA-193	PRO-219
VAL-194	PRO-247
VAL-194	VAL-248
THR-195	PRO-247
ASN-196	PRO-247
GLU-199	PRO-247
LYS-240	ARG-406
GLU-244	ARG-406
ARG-278	GLU-431
**H-Bond interactions**	
**KIF5B Residue**	**Tau Residue**
ASN-152	ASP-252
LYS-240	ARG-406
LYS-240	SER-409

GLU-244	HIS-407
ALA-251	ASN-410
LYS-256	THR-414
**Salt-Bridges**	
**KIF5B Residue**	**Tau Residue**
LYS-141	ASP-252
LYS-141	ASP-421
LYS-141	GLU-36
ASP-249	ARG-406
LYS-256	ASP-421
LYS-281	ASP-421
LYS-281	GLU-36
ARG-284	GLU-431

Interacting residues at the ATP binding domain of Kif5b are highlighted in dark green, and those near the ATP binding domain are highlighted in light green.
